# Simvastatin combined with bone marrow mesenchymal stromal cells (BMSCs) improve burn wound healing by ameliorating angiogenesis through SDF-1α/CXCR4 pathway

**DOI:** 10.22038/ijbms.2020.39782.9465

**Published:** 2020-06

**Authors:** Javad Mohajer Ansari, Parisa Ramhormozi, Ronak Shabani, Hamidreza Pazoki-toroudi, Abazar Yari, Mahmood Barati, Mostafa dahmardehei, Azar Babakhani, Maliheh Nobakht

**Affiliations:** 1 Department of Anatomical Sciences, School of Medicine, Iran University of Medical Sciences, Tehran, Iran; 2 Physiology Research Center and Department of Physiology, Faculty of Medicine, Iran University of Medical Sciences, Tehran, Iran; 3 Department of Anatomy, Faculty of Medicine, Alborz University of Medical Sciences, Karaj, Iran; 4 Dietary Supplements and Probiotics Research Center, Alborz University of Medical Sciences, Karaj, Iran; 5 Deparment of Medical Biotechnology, Faculty of Allied Medicine, Iran University of Medical Sciences, Tehran, Iran; 6 Burn Research Center, Iran University of Medical Sciences, Tehran, Iran; 7 Anti-Microbial Resistance Research Center, Iran University of Medical Sciences, Tehran, Iran

**Keywords:** Angiogenesis, Bone marrow mesenchymal, stromal cells, C-X-C motif chemokine, receptor 4, Stromal drive factor-1α, Simvastatin, Wound healing

## Abstract

**Objective(s):**

Chemokines are wound mediators that promote angiogenesis during wound healing. We hypothesized that Simvastatin in combination with the bone marrow mesenchymal stromal cells (BMSCs) improve burn wound healing by ameliorating angiogenesis via SDF-1α/CXCR4 pathway.

**Materials and Methods:**

Under general anesthesia, deep partial-thickness burns were created on the inter-scapular area of 48 male rats. Study groups were administrated with petroleum jelly (Simvastatin Vehicle), a single dose of intradermal BMSCs (1×10^6^), topical Simvastatin (0.5 mg/kg) daily and combination of BMSCs and Simvastatin for 14 days. In this study, we used MTT assay, *in vivo* and *in vitro* wound closure, H&E and Trichorome staining, immunohistochemistry (IHC), real- time PCR, Western blot and tube formation assay.

**Results:**

A significant improvement in wound closure percentage, epithelial thickness, collagen remodeling, and up-regulation of stromal cell-derived factor 1 alpha (SDF1α), C-X-C chemokine receptor type 4 (CXCR4), protein kinase B (AKT), and phosphatidylinositol 3- kinase (PI3K), as well as CD31 and vascular endothelial growth factor (VEGF) expression were observed after treatment with simvastatin, BMSCs and combination of them compared to the vehicle group. However, the co-treatment group revealed considerable superiority in examined factors. BMSCs treated with Simvastatin showed the highest viability in the concentration of 0.5 and 1 Nanomolar (nM). Increment in proliferation and capillary vessels formation of BMSCs was observed in the 0.5 nM and 1 nM concentrations of Simvastatin *in vitro*.

**Conclusion:**

Treatment of deep partial-thickness of burns with co-treatment of BMSCs and Simvastatin resulted in improved burn wound healing through up-regulating of SDF-1α/CXCR4 pathway.

## Introduction

Burn is an injury that causes disruption in integrity and normal function of affected tissue (1). Chemicals, radiation, heat, cold, friction, and electricity are the main causes of burn injury. At the time of insult, numerous pathways are activated to regenerate tissue integrity. Therefore, the process of burn wound healing is a multifactorial, multiphase and complex process (2). One of the most accepted approaches in the burn wound healing care is the speed up in wound healing time, which decreases patient morbidity and mortality. Despite new findings in wound care, acceleration of wound healing remained as a clinical challenge; therefore, new treatment strategies such as gene therapy (3), growth factor therapy (4), and stem cells therapy (5) are developing. Stem cells can participate in wound healing processes by two side by side strategies: A) Contributing in tissue repair through cell shortage compensate, B) Secretion of many essential factors including growth factors and cytokines (6). 

Bone marrow mesenchymal stromal cells (BMSCs) are multipotent stem cells, which are capable of self-renewal and differentiation to various cell types (7). Furthermore, many therapeutic capacities of BMSCs depend on paracrine factors, which attribute to antiapoptotic, angiogenic and mitogenic activities (8). In this regard, BMSCs can speed up wound healing process by multiple strategies such as re- epithelization via differentiation into keratinocytes, participating in derm layer rebuilding by differentiation into fibroblast, improvement in angiogenesis in skin dermis through differentiation to endothelial cells (9) and releasing cytokines as well (10). Chemokines act as key regulators involved in various wound healing processes such as angiogenesis promotion in inflammation and proliferation phases (11). SDF-1α/CXCR4, a chemokine pathway consists of stromal cell-derived factor 1 alpha (SDF-1α) and its chemokine receptor type 4 (CXCR4), plays a key role because not only has an effect on cell migration and proliferation but also affect angiogenic activity (12, 13). SDF-1α/CXCR4 pathway convinces signalling of protein kinase B/ phosphatidylinositol 3-kinase (AKT/PI3K) and following that, up-regulates vascular endothelial growth factor (VEGF) (14). Furthermore, SDF-1α/CXCR4 signalling regulates PI3K/Akt/ endothelial nitric oxide synthase (eNOS) pathway that results in diminished endothelial cells apoptosis (15). Interestingly, among other MSCs, the highest SDF-1α gene expression belongs to BMSCs, which makes them a preferable source for stem cell therapy, either locally or systematically (16). 

Simvastatin is a reductase inhibitor that belongs to the statins family. It is able to reduce plasma cholesterol level through 3-hydroxy-3-methylglutaryl -coenzyme A (HMG- CoA) inhibitor. Generally, statins are being used for the management of hypercholesteremia (17). Besides the main role, Simvastatin has numerous pleiotropic effects such as, angiogenesis, cell migration, and cytokines expression alteration (18, 19). SDF-1α/CXCR-4 is one of the signalling pathways that is influenced by Simvastatin. Simvastatin is able to increase the expression of not only SDF-1α, but also its receptor, CXCR4. Therefore, co-treatment of Simvastatin and BMSCs may have synergic effect on the wound healing process through SDF-1α/CXCR-4 signalling pathway (20). It can be of the interest approach in the treatment of burn wounds. Given the background, in this study we aimed to assess the additive therapeutic effects of BMSCs and Simvastatin combination in burn wound healing. We hypothesize that this multicomponent therapy leads to up-regulation of SDF-1α/CXCR4 pathway, which subsequently promotes the process of wound healing through angiogenesis. 

## Materials and Methods

***Animals***

Forty eight male Wistar rats, weighing between 200 to 250 g were obtained from the Animal Laboratory of Iran University of Medical Sciences (IUMS). They were housed and maintained in polypropylene cages under controlled environmental conditions (Light-dark cycle: 12 hr/12 hr, temperature: 25±1 ^°^C and humidity: 60±5%). Seven days before burn induction, they were housed in cage individually for reconciliation. Animal protocols used in this study were reviewed and approved by the Ethical Committee of Iran University of Medical sciences. Whole of project was performed in 12 month.

***Wound creation, dressing and treatment***

Rats were separated accidentally into four groups (n=12) including: Vehicle (animals were treated with petroleum jelly), BMSCs group (animals were received single dose of BMSCs using intradermal injection), Smv group (animals were administrated with topical Simvastatin) and BMSCs & Smv (animals were treated with combination of BMSCs and Simvastatin). Before surgery, the hairs on the back of the rats were shaved by using a razor under anesthesia with Ketamine (15 mg/kg)/ Xylazine (2.0 mg/kg), and the shaved area was disinfected in 2 steps with 70% Isopropyl alcohol and Povidone-iodine. Rats were laid ventrally and a rectangular iron lid connected to a temperature-controlled super soldering station (RX-711AS- Goot, Hiroshima, Japan) was steadily applied on the interscapular area for 10 sec, at 120 ˚C to induce a deep partial thickness in a rectangular shape in 1.5×1.5 cm^2^ size. Under similar conditions, each wound was covered with a transparent dressing (coloplast, Tehran, Iran). Topical Simvastatin was prepared as previously described (21). Simvastatin (0.5 mg/kg) in Petroleum jelly or Petroleum jelly alone was used for wound dressing in each day. In 14 days after burn injury, rats were sacrificed and skin samples were collected for histology and molecular evaluation.

***BMSCs isolation, culture, characterization and application***

BMSCs were isolated and cultured based on previously described method (22). At a glance, bone marrow of femur and tibia of the rat were isolated and incubated for 48 hr with 90% DMEM and 10% fetal bovine serum (FBS) at conditioned environment (37 ^°^C, 95% humidity and 5% CO_2_). After 48 hr, the culture medium changed with fresh medium for the elimination of hematopoietic stem cells. Every 3 days, medium was changed according to the color change of medium. Since cell population reaches to 70%-80% confluency, cells were subcultured in ratio of 1:3. Expression of CD 105, CD90 (positive markers) and CD45, CD44 (negative markers) were determined by flow cytometry using antibody (FITC labeled) against them (BD Biosciences, San Jose, CA). Intradermal injection of BMSCs (1 ml of phosphate-buffered saline (PBS) containing 1×10^6^ BMSCs) was performed in the combined (BMSCs & Smv) and BMSCs groups. 

***Multipotent differentiation***

To determine differentiation potential of BMSCs, cells from passage 3 were placed in the basic medium (DMEM, 10% FBS, 1% penicillin, Invitrogen) at 6 well plates and differentiation medium was added to each plate. For chondrogenic differentiation, cells were cultured with the basic medium, which contained 50 M ascorbic acid, 0.1 mM dexamethasone, 10 ng/ml TGF, and 40 g/ml and 100 g/ml sodium pyruvate (Sigma-Aldrich) for 21 days. For osteogenic differentiation, 10 nM dexamethasone, 50 mg/ml ascorbic acid, and 10 mM β glycerophosphate (Sigma-Aldrich) were added to the basic medium. Adipogenic differentiation was obtained with a 100 nM dexamethasone and 0.1 mM indomethacin (Sigma-Aldrich) as a differentiation medium. Differentiated cells were stained with toluidine blue (chondrocytes), Oil red O (adipocytes) and Alizarin Red (osteocytes). 

***In vitro cell viability***

MTT assay was used for measuring cell proliferation and survival rate. Cells were seeded at an initial density of 5×10^3^ per well in 96 well plates for 24 hr. After 24 hr, cells were treated in the same manner with different Simvastatin dose (0.1 nM, 0.5 nM, 1 nM, and 10 nM) for the 72 hr. After exposure with different concentration of Simvastatin, 200 µl of CM containing 10% MTT (thiazolyl blue, Sigma) solution was added to each well and incubated at 37 ^°^C for 3 hr in darkness, and then media was removed and 200 µl DMSO was used to solubilize formazan crystals. The optical density was determined by the ELISA reader at 570 nm.

***In vitro wound healing***

BMSCs were seeded into 6 well plates at a density of 0.3 × 10^6^ cells per well. At 95% confluency, Scratch assay was performed with a p-200 pipet tip to create a straight line of scratch, followed by triple washes with PBS to remove detached cells. Scratched monolayers of BMSCs were treated with medium containing various concentrations of Simvastatin or without Simvastatin as the control. Percentage of wound closure was determined over a period of 24, 48, and 72 hr. Six fields per each time point were photographed under an inverted microscope (OlympusAX70) and a Canon digital camera (Power shot SX30 IS). Percentage of wound healing area in images was determined with the ImageJ version 1.44 (NIH, Bethesda, MD). Images were analyzed based on method described by Dr. Kees Straatman (Advanced Imaging Facilities, University of Leicester, Leicester, UK).

***In vivo wound healing***

*In vivo* wound healing rate was determined by comparing wound area in 0, 3, 7, 10, and 14 days after wounding the initial area. Images were taken by a Canon digital camera (Power shot SX30 IS) and wound area images were recorded and analyzed using ImageJ software. The wound healing rate was calculated as follow:

[Area of original wound- area of initial wound)/ Area of initial wound] ×100

***In vitro angiogenesis assessment***

Analysis of BMSCs capillary formation was performed using the collagen type I-coated flask. BMSCs were cultured with conditioned medium (contain desire Simvastatin concentrations) for 48 hr. Then, cells were trypsinized and 5 × 10^3^ cells were suspended and plated onto the collagen type I gel. Cells were observed to form capillary structures. The average of tube length was calculated in the groups that were treated with Simvastatin (23).

***Assessment of wound breaking strength***

Animals were sacrificed with an overdose of Ketamine and Xylazine on day 14 post-wound creation, and skin of dorsal surface including wound area (1 cm in width and 4 cm in length) were removed. The maximum load (breaking strength) tolerated by wounds was measured blindly on coded samples using a calibrated tensiometer (SPM20 model, ASTM D882-02 from F2150-02, Load Cell, Korea) as described previously (24). The ends of the skin strip were pulled at a constant speed (20 mm/min), and breaking strength was expressed as the wound breaking strength (Newton) before the separation of wounds.

***Histology staining***

On day 14 after wounding, tissue specimens were removed and fixed in Bouin fixative for at least 48 hr at room temperature. Following histological samples embedding in paraffin, the samples were cut and mounted on slides (5- µm-thick) and were then stained with H&E (to assess epithelial thickness) and Masson’s trichrome (to determine remodeled collagen content). All images were evaluated by the observer without knowledge of the previous treatment. The ImageJ was used for evaluation of epithelial length (µm) and collagen density (Collagen intensity percentage) according to methods in the literature concerning wound healing in an experimental model.

***In vivo angiogenesis assessment***

To determine *in vivo* angiogenesis, immunohistochemistry (IHC) for CD31 and VEGF was performed. Tissue sections (6 µm) were prepared for immunostaining after deparaffinization, rehydration, antigen retrieval (was performed using Tris-EDTA) and antibody blocking (incubating the section in 2% [v/v] normal goat serum in PBS for 20 min). Endothelial cells were detected with the anti-rat CD31 antibody (ab24590) as primary antibody at a 1:500 dilution (overnight incubation) and VEGF antibody (ab46154). Anti-goat FITC (ab6840) was used as the secondary antibody in 1:2000 dilution (1 hr incubation). ImageJ software was used for calculating the percentage of the fluorescent area. 

***Real time qRT-PCR***

For real-time polymerase chain reaction (PCR), wound area was obtained and stored in -80 ˚C until RNA was extracted using TRI reagent (Sigma Aldrich-Germany). RNA purity and concentration of the samples were analyzed using a NanoDrop (Thermo Scientific- United States). Complementary DNA was generated with TAKARA complementary DNA synthesis kit (Otsu, Japan). Real-time PCR was conducted with complementary DNA and TAKARA SYBR Green (Otsu, Japan) in a Bio-Rad iCycler. The candidate primers used for quantitative RT-PCR are listed in Table 1.

***Western blot analysis***

Tissue samples were lysed with RIPA buffer (Santa Cruz). Tissue lysates were centrifuged at 12,000 × g for 10 min at 4 ^°^C, and the supernatants were collected and stored in -80 ˚C until extracted proteins were used for western blot. Samples (40 µg protein) were boiled for 5 min at 95 ^°^C and were separated by 10% or 8% SDS-PAGE (based on protein molecular weight). The PVDF membranes were incubated with anti-SDF1α antibody (ab9797), rabbit anti-pan-AKT antibody (ab8805), or anti-GAPDH antibody (ab245357) and detected with horseradish peroxidase–conjugated goat anti-rabbit IgG (Bio-Rad, Hercules, CA).

***Statistical analysis***

Statistical analysis was performed using Prism software (GraphPad Software, La Jolla, CA). One - way analysis of variance (ANOVA) was used for comparison between groups. The paired t-test and two-way ANOVA was used to determine the difference between Simvastatin concentrations in tube formation assay and *in vivo* wound closure, respectively. *P*<0.05 was considered significant and data are expressed as mean±SEM. 

## Results

***Standardization of burn severity***

To create consist and uniform deep partial thickness wounds, models were inflicted in different temperatures of 50, 70,100, and 150 ˚C in 10 sec. Since stasis level of burn is complete 3 days after burning, burn samples were collected at this time. Model confirmation is performed by H&E staining. In regard to the depth of the damage, finding in 150 ˚C was in favor of the deep partial thickness of burn. Representative histological images are shown in Figure 1-s of supplementary file. 

***Tri-lineage differentiation and characterization of BMSCs surface markers***

**Figure 1:**
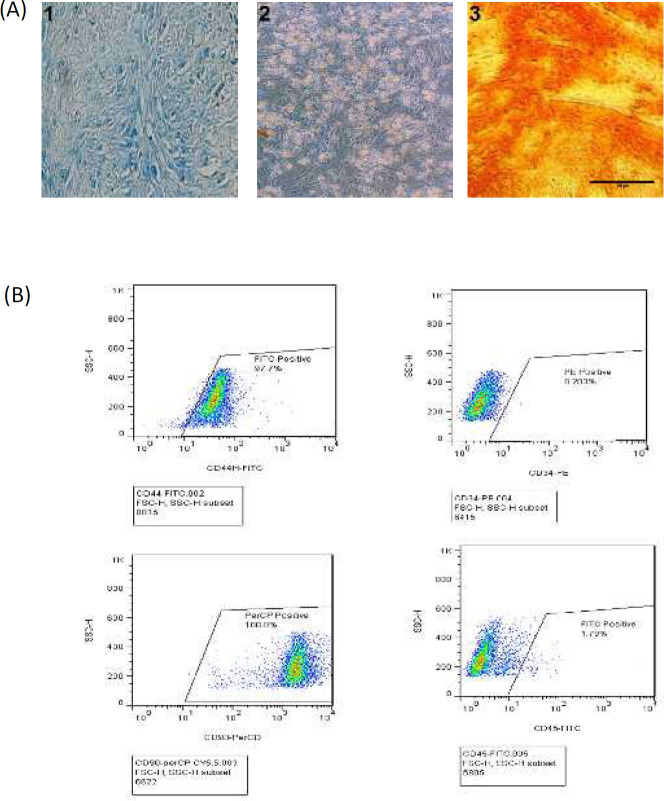
Identification and differentiation of BMSCs. A) BMSCs were cultured in chondrogenic, adipogenic or osteogenic differentiation mediums. Cells were stained with Alcian blue for chondrogenesis (1), Oil Red O for adipogenesis (2) and Alizarin red for osteogenesis differentiation (3). B) BMSCs from passage 3 were positive for mesenchymal cell markers; CD44-FITC and CD90-PE, and negative for hematopoietic markers; CD45- FITC and CD34-PE

Tri-linage differentiation (osteogenic, adipogenic, and chondrogenic lineages) was approved by their especial histology stains (Figure 1A). BMSCs from the passage 3 expressed immuno-positivity for CD90 and CD44 (more than 95%) and negativity for CD45 and CD34 (less than 2%; Figure 1-B). The morphology of cells were presented in Figure 2-s of supplementary file data. 

***Biomechanical strength of the wounds***

**Figure 2:**
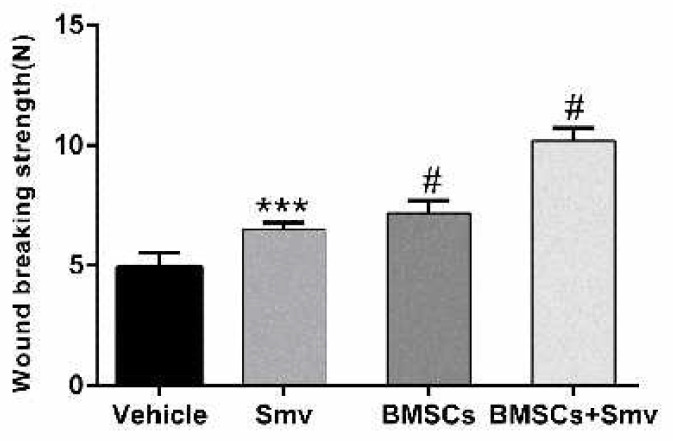
Effect of co-treatment of BMSCs & Smv on wound breaking strength in burn wounds

Vehicle, BMSCs, Smv and co-treated (BMSCs & Smv) groups were euthanized on day 14 post-wounding to determine the wound breaking strength (df: 3, 16; *F*=98.8; *P*<0.0001). Breaking strength in BMSCs & Smv (10.21±0.52 N), BMSCs (7.188±0.5317 N) and Smv (6.502±0.2863N) groups showed significant increase compared to vehicle group (4.944±0.5861 N). There was a significant improvement in biomechanical feature of the treated wounds compared to the vehicles. These data indicate that BMSCs & Smv treatment has an additive effect on biomechanical feature of wounds (Figure 2).

***Gross appearance of wounds after treatment with BMSCs and Smv***

**Figure 3:**
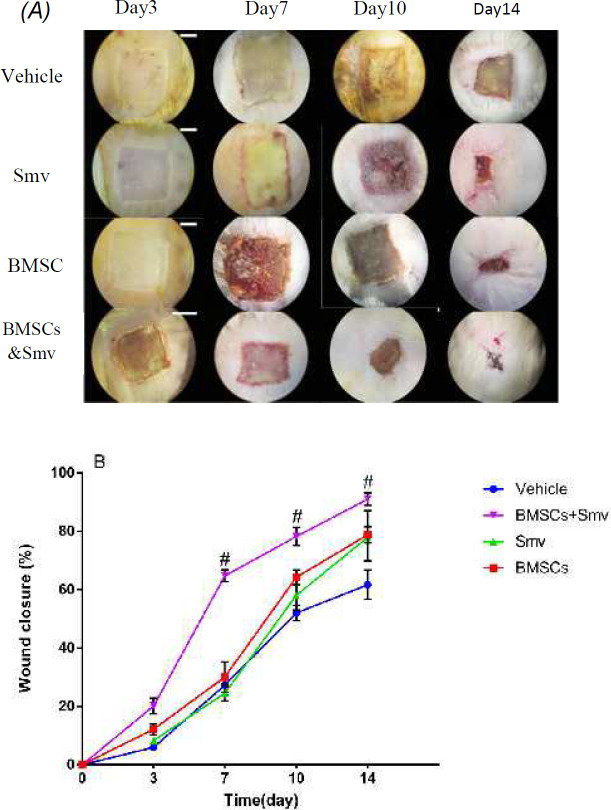
A) Gross appearance of wound contraction in different days after various treatments. B) Wound closure percentage of deep partial thickness of burn in 3, 7, 10, 14 days after wounding. Groups had been compared versus vehicle in each day. Data are given as mean±SD. * represent *P<*0.05, ** for *P<*0.001 and # stands for *P<*0.001

Gross appearance of wounds were measured after 3, 7, 10 and 14 days of wounding in Smv, BMSCs, vehicle or BMSCs & Smv groups. On day 14, BMSCs & Smv-treated wounds had more than 90% wound closure (91.31±3.87%), whereas wound closure percentage was lower in BMSCs (81.75±8.26%) and Smv (87.89±3.45%) groups. Besides, in the vehicle group, wound healing was less than the others (60.42±6.72%). Macroscopic view of wounds and related data are illustrated in Figure 3. 

***Epidermis regeneration and collagen remodeling***

**Figure 4:**
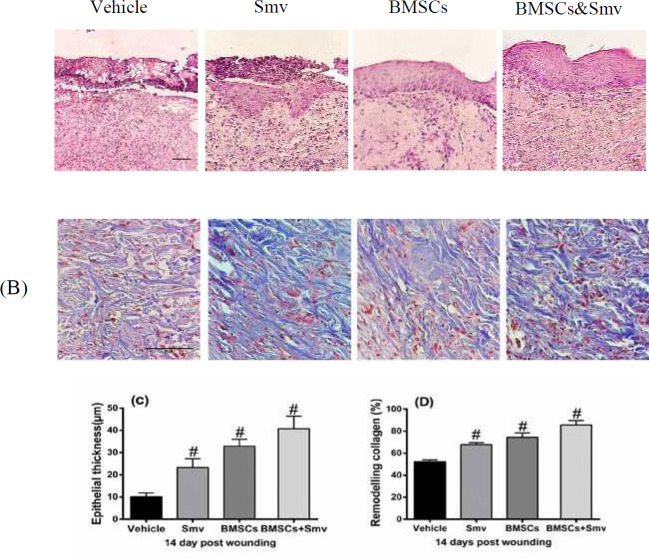
Photomicrograph and histological analysis of the burn wounds

H&E and Masson’s trichrome staining were used to evaluate epidermal thickness (df: 3; *F*=94.71; *P*<0.0001) and collagen remodeling (df: 3; *F*=121.6; *P*<0.0001), respectively. BMSCs & Smv group had higher epidermal thickness (40.78±5.572 µm) than the others, whereas BMSCs (32.89±3.054 µm) and Smv (23.32±3.951 µm) have a thinner thickness but showed an improve in epithelialization versus vehicle (10.15±1.676 µm). Intensity of blue color in the Figure is equivalent to remodeled collagen content. The amount of remodeled collagen was highest in the BMSCs & Smv group (85.53±4.183%), which followed by the BMSCs (74.53±3.834%) and Smv (67.87±1.711%) groups. Lowest quantification amount of collagen fibers belongs to the Vehicle group (52.22±1.791%). Photomicrograph and data of H&E and Masson’s trichrome are presented in Figure 4.

***In vitro cell viability***

**Figure 5:**
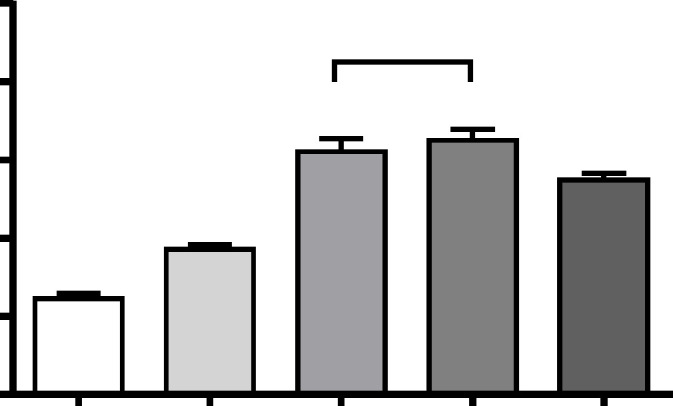
Effect of different concentrations of Simvastatin on BMSCs viability and proliferation at 72 hr. The viability of BMSCs was not affected in any tested concentrations. The promotion in proliferation was dose-dependent and occurred in 1 nM and 0.5 nM concentrations of Simvastatin. Values are expressed as mean±SD of eight determinations.*P<0.0001 vs control. BMSCs: Bone marrow mesenchymal stromal cells

 Effect of Simvastatin on BMSCs viability was tested with different doses of Simvastatin (0.1 nM, 0.5 nM, 1 nM, and 10 nM) at 72 hr after incubation (df: 8; *F*=1443; *P*<0.0001). The results showed that the viability of BMSCs was not significantly affected by different Simvastatin doses compared to the control. In this study, 0.5 nM and 1 nM Simvastatin were selected for tube formation assay due to attaining to the maximum proliferation compared to the other doses (Figure 5). 

***In vivo Angiogenesis assessment***

**Figure 6:**
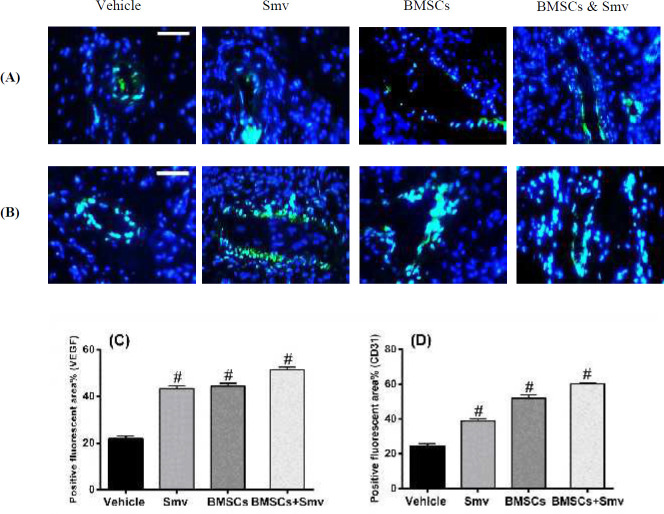
Effects of vehicle, Simvastatin, BMSC or BMSC & Smv tretment on CD31 and VEGF expression A,B) Merged picture of immunostaining illustrates VEGF (A) and CD31 (B) expression in vehicle, Simvastatin, BMSCs or in combination treatment at the wound area after 14 days. Scale bars = 100 µm. C,D) Percentage of positive felurocent area (CD31 and VEGF) quantified as described in Materials and Methods. # *P<*0.0001 compared to vehicle group. BMSCs: Bone marrow mesenchymal stromal cells

We performed IHC for CD31 (df: 3; F=713.1; *P*<0.0001) and VEGF (df: 3; F=652.9; *P*<0.0001) detection to determine the effects of Simvastatin and BMSCs on *in vivo* angiogenesis. IHC for VEGF revealed that the highest positivity belonged to the BMSCs and Simvastatin treatment group (51.41±1.17%) followed by Simvastatin (43.36±1.27%) and BMSCs (44.35±1.28%) and vehicle (21.87±1.15%). CD31 positivity in combined treatment group (BMSCs and Simvastatin) was significantly higher than the other group (60.24±0.65%). However, monotherapy with BMSCs (52.01±1.87%) or Simvastatin (38.88±1.37%) had a positive effect on CD31 expression but not as much as combined treatment. Percentage of the positive fluorescent area in the vehicle group was lower than the other groups (24.20±1.58%). Merged pictures and quantified data for VEGF and CD31 were represented in Figure 6. CD31, VEGF mono fluorescence and DAPI-labelled nuclei pictures are shown in the supplementary file (Figure 3-s). 

***Tube formation assay***

**Figure 7:**
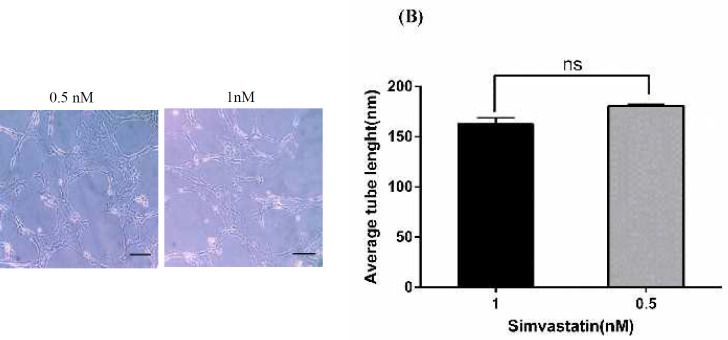
Tube formation assay. Simvastain stimulates tube formation of BMSCs plated on type I collagen

BMSCs were plated on type I collagen-coated wells for 48 hr after pretreated with 0.5 and 1 nM concentrations of Simvastatin (t=10.84; F=8; *P*<0.0001). Both concentrations of Simvastatin enhanced tube formation. Average tube length for 0.5 nM and1 nM was 162.56±6.22 µm and 180.73±1.75 µm, respectively. Although the average tube length of 0.5 nM concentarion is slightly higher but difference between 0.5 nM and 1 nM is insignificance (Figure 7A, B). 

***Wound healing assay***

**Figure 8:**
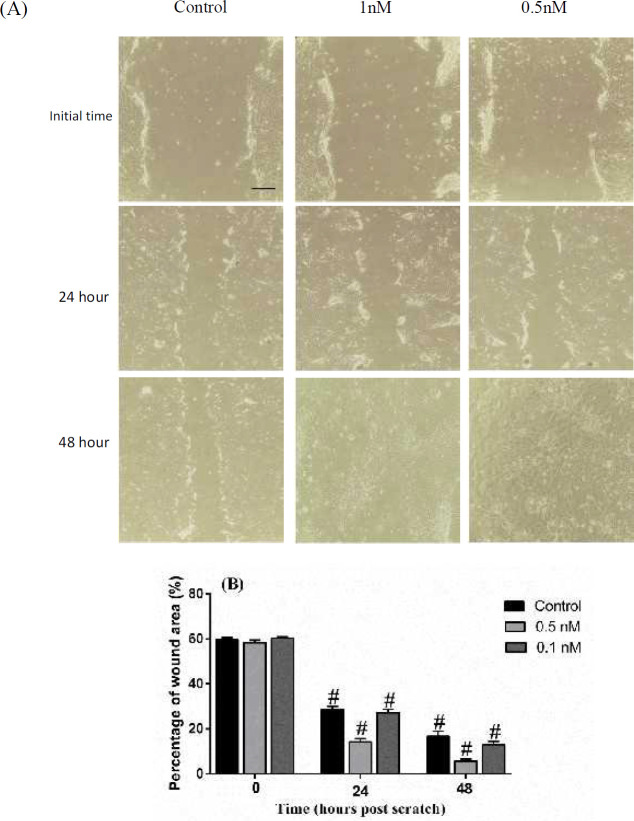
A) Exposure to Simvastatin increases BMSCs migration in in vitro scratch assay. A confluent BMSCs monolayers were grown in 6-well plates containing 1 nM and 0.5 nM concentration of Simvastatin (scale bar=50 µm). Cell scratches were imaged at 0, 24 and 48 hr after the scratch using invert microscope (magnification 20X). B) The data represent a mean±SD of wound healing percentage area. # *P<*0.0001 vs control (n=8). BMSCs: Bone marrow mesenchymal stromal cells

*In vitro* wound healing was evaluated in no exposition or exposition to desire concentration of Simvastatin (1 nM and 0.5 nM) in 0,2 4, and 48 hrs (df: 8; F=1443; *P*<0.0001). In 24 hr after wound creation, percentage wound closure area was (14.27±1.52%) in 0.5 nM and (27.14±1.64%) in the 1 nM dose of Simvastatin whereas (59.68±0.99%) in control wound, at the same time. After 48 hr of initial time, further decrease was observed in wound area of 0.5 nM (5.68±0.96%) and 1 nM (12.98±1.42%) treated cells versus control (16.70±2.32%). Scratch assays revealed that BMSCs treated with Simvastatin accelerate the wound closure, while the control group of BMSCs was unable to elicit the same effect (Figure 8A, B). The edges and final outline images were shown in supplementory data file (Figure 4-s and 5-s).

***mRNA expression level***

**Figure 9:**
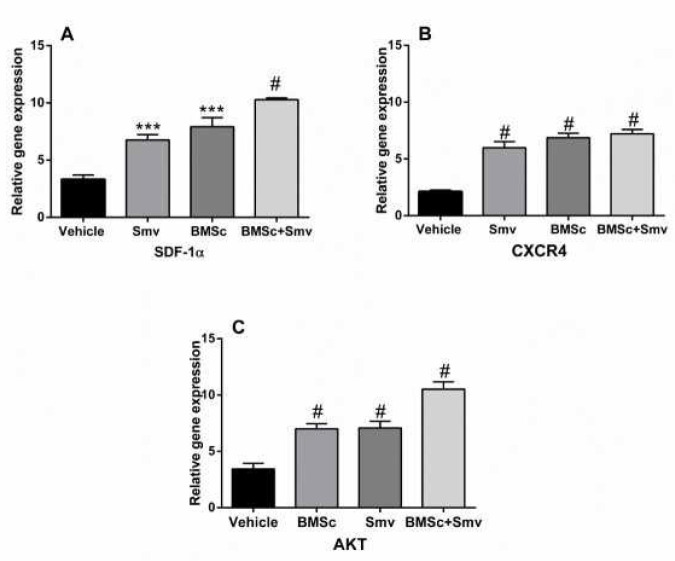
Gene expression of SDF1α, CXCR4, and AKT. Relative to the vehicle, animals were treated with Simvastatin, BMSC or BMSC & Smv over-expressed mRNA of SDF1α, CXCR4, and AKT. Up-regulation of mRNA was higher in the combined group compared to other treated groups. Threshold cycles were normalized based on the reference gene GAPDH groups. Data are represented as mean±SD (n=3). # *P<*0.0001 versus vehicle. SDF1α: Stromal cell-derived factor 1 alpha

Real time PCR was used to assess mRNA expression level of SDF1α (df: 2.03; F=10.19; *P*<0.0001), CXCR4 (df: 3; F=31.03; *P<*0.0001), and AKT (df: 3; F=81.72; *P*<0.0001) genes. Figure 9 illustrates that recruitment of Simvastatin, BMSCs or co-tretment of them can cause the wound healing through modulations of the gene expression. Although, combined therapy with BMSCs and Simvastatin has an additive effect on expression of SDF-1α, CXCR4, and AKT compared to the other groups. 

***Western blot analysis***

**Figure 10:**
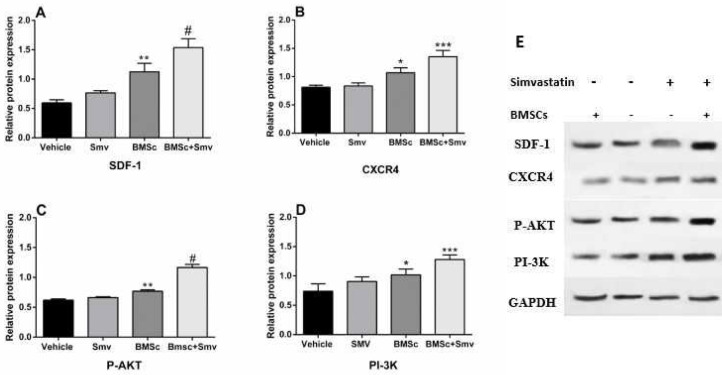
Western blot analysis on the protein expression of SDF-1-α (A), CXCR4 (B), PAKT (C), and PI3K (D) and western blot bands (E). Skin samples of burn lesions were prepared at 14 days after burn induction. Combined treatment with Simvastatin and BMSCs promoted significant up-regulation in SDF-1α and CXCR4 as well as AKT and PI3K. * represent *P<*0.05, ** for *P<*0.001 and # stands for *P<*0.001

Western blot analysis for SDF1α (df: 3; F=43.65; *P*<0.0001), CXCR4 (df: 3; F=31.03; *P*<0.0001), PAKT (df: 3; F=197.8; *P*<0.0001) and PI3 (df: 3; F=16.48; *P*<0.0001) was performed with protein extrcted from skin tissues to test whether treatment with vehicle, Simvastain, BMSCs or combination of Simvastain and BMSCs affect the level of proteins involved in wound healing on day 14. There was a significant difference in desire protein expression in Simvastatin, BMSCs, and combined groups versus vehicle group. However, highest protein level epression belongs to co-treatment group (Figure 10). 

**Table 1:** Designed primers sequence used for real-time PCR

Genes	Forward seq. (5' to 3')	Reverse seq. (5' to 3')
SDF1 CXCR4 AKT GAPDH	CTCCAAACTGTGCCCTTCAGA CAGCAGGTAGCAGTGACCCT GCCACGGATACCATGAACGA AGTGCCAGCCTCGTCTCATA	TCCTTTGGGCTGTTGTGCTTA AGGGTTCCTTGTTGGAGTCAT TTGAGGAGGAAGTAGCGTGG GAGAAGGCAGCCCTGGTAAC

SDF1α: Stromal cell-derived factor 1 alpha, Cxcr4: C-X-C chemokine receptor type 4, AKT: Protein kinase B, GAPDH: Glyceraldehyde 3-phosphate dehydrogenase

BMSCs: Bone marrow mesenchymal stromal cells

Male adult wistar rats were treated with Vehicle, BMSCs, Smv or BMSCs & Smv combination. Animals were sacrificed on Day 14 and specimens breaking strength were determined by a tensiometer as described in methods & materials. Statistical analysis was performed using ANOVA (mean±SE). ***P<0.001, # *P<*0.0001 vs vehicle group. BMSCs: Bone marrow mesenchymal stromal cells, Smv: Simvastatin

A) Hematoxylin & eosin (H&E) staining to compare epithelial thickness (µm) after treatment with vehicle, BMSCs, Smv or BMSCs & Smv in 14 days after wounding. B) Masson’s trichrome staining revealed collagen intensity percentage (%) in the same group. Scale bars=50 µm. C,D) Quantitative data of epithelial thickness and collagen content. Values are analyzed and reported by One-way ANOVA and means±SD, respectively. # stands for *P<*0.001. BMSCs: Bone marrow mesenchymal stromal cells, Smv: Simvastatin

Smv: Simvastatin, VEGF: Vascular endothelial growth factor

A) BMSCs were cultured with medium contain different concentraions of Simvastatin (1 nM and 0.5 nM) for 24 hr and then cells were transfered to collagen-coated wells. Photographs were taken after 48 hr. Bar= 50 µm. B) Paired t-test procedure was used to determine difference between goups. There is no significant difference in average of tube formation between concentrations. Average tubes length are given as mean±SD

BMSCs: Bone marrow mesenchymal stromal cells

CXCR4: C-X-C chemokine receptor type 4, AKT: Protein kinase B, GAPDH: Glyceraldehyde 3-phosphate dehydrogenase, BMSCs: Bone marrow mesenchymal stromal cells

SDF1α: Stromal cell-derived factor 1 alpha, CXCR4: C-X-C chemokine receptor type 4, PAKT: Phosphorylated protein kinase B, PI3K: Phosphoinositide 3-kinase, BMSCs: Bone marrow mesenchymal stromal cells

## Discussion

Augmentation of angiogenesis is critical to accelerate the wound healing process. CXC family of Chemokines promotes angiogenesis during inflammatory and proliferation phases of the wound healing (11). This study demonstrates that combination treatment of Simvastatin and BMSCs improves the process of wound healing through SDF-1α/CXCR4 signalling pathway. There was an additive improvement in angiogenesis following co-treatment of BMSCs with Simvastatin compared to the monotherapies. Stem cell therapy and pharmacotherapy are two main strategies to ameliorate angiogenic activity (25). BMSCs are promising candidate for the stem cell therapy, which can involve in the wound healing process by improvement in some activities such as acceleration of the wound closure, reduction of reactive oxygen species at the injury site, enhancement of Neo-vascularization, over expression of VEGF, and improvement in SDF-1α secretion as well (26). Using of Simvastatin as a pharmacological approach also increases angiogenic activity through modulation of VEGF protein secretion, CD31 expression increasment, regulation of differentiation and apoptosis in endothelial cells, as well as up-regulation of SDF-1α/CXCR4 and PI3-kinase/Akt pathways (13). To further elaborate the effect of SDF-1/CXCR4 pathway on angiogenesis, we investigated PI3K/AKT expression levels, which have been previously shown to be significantly correlated with angiogenesis. In this study, we showed that three treatments of Simvastatin, BMSCs, and simvastatin & BMSCs increase the expression of PI3K/AKT alongside SDF-1/CXCR4. Interestingly, combination treatment of Simvastatin and BMSCs indicated higher protein expression than the others. SDF-1α/CXCR4 pathway exists in various stromal cells and injured tissue. Up-regulation of SDF-1α following injury in several organs and various regeneration processes has been reported in many studies. In the process of wound healing, SDF-1/CXCR4 pathway impresses the differentiation of epithelial like cell and endothelial cells for re-epithelialization and angiogenesis, respectively. Moreover, wound contraction and connective tissue production is facilitated by SDF-1/CXCR4 pathway through myofibroblasts and fibroblasts activation (27). Multicomponent therapy of Simvastatin and SDF-1α showed enhancement in angiogenesis. One of the most known sources of SDF-1α is BMSCs, which naturally express SDF-1α as a chemotactic factor for maintaining hematopoietic stem cells in bone marrow (13). Our findings provide conclusive support for higher expression of SDF-1α and CXCR4 in BMSCs-treated group. Recently, we showed that CXCR4 blockage with AMD3100 can cause delay in wound healing in rat (28). SDF-1α up-regulates PI3K/Akt signalling, which stimulates the phosphorylation of eNOS and subsequently leads to increment of endothelial nitric oxide (NO) production (15). NO can elevate proangiogenic cytokines expression of MSCs to promote the angiogenic response. NO also promotes angiogenesis through mediating the activity of VEGF (29). In confirmation to our results, IHC revealed a raise in angiogenic factors expression; VEGF and CD31 in combined treated group compared to the other groups. It has also been demonstrated that combination therapy has an additive effect including wound closure, re-epithelialisation, and collagen remodelling compared to other treatments. Besides, we have considered the influence of Simvastatin and BMSCs combination treatment on wound integrity. Wounds that undergo co-treatment of Simvastatin and BMSCs showed more enhancement in solidity (regeneration of tissue strength) and elasticity (increase collagen deposition) in comparison with the monotherapies. Collagen is the predominant dermis protein that has a key role in anatomic integrity of wounds. Moreover, collagen remodelling supports the re-epitalization of wound by providing a proper bed for epithelial cells (30, 31). Treated wounds with the three treatments showed an increment of wound contraction and epithelialisation compared to the vehicle group, although combination treatment accelerates the wound closure more than individual treatments. Our result is consistent with findings of the past studies by Kwon *et al.* and Bitto and co-workers, which reported that diabetic wound treatment with either Simvastatin or BMSCs improves the wound healing process through enhancement of collagen synthesis, breaking strength and epithelialisation (24, 32).

Our *in vitro* investigation showed that Simvastatin facilitates BMSC proliferation, migration, and tube formation in Nano molar concentrations, which coincide with the pervious findings (9). However, adding higher concentration of Simvastatin to the culture medium causes apoptosis, suggesting that the dosage of Simvastatin should be carefully selected for *in vitro* studies (33).

## Conclusion

Our data demonstrate that combined treatment of Simvastatin and BMSCs improves wound healing possibly through promoting SDF1/CXCR4 pathway. This combination therapy mediated angiogenesis, which maybe the reason of improved healing outcome after the burn.
